# Filling Cavities of Decay in Proximal Surfaces

**Published:** 1874-02

**Authors:** Marshall H. Webb


### ARTICLE II.

### *Filling Cavities of Decay in Proximal Surfaces.*

BY MARSHALL H. WEBB, D. D. S.

In the mouth of civilized man we find an acid breaking down the enamel prisms and exposing the dentine, with its tubuli, when the progress of disintegration seems to be accelerated by the presence of *Leptothrix buccalis*, which are thought to have some influence in the causation of caries.

As this attack is usually made upon the proximal surfaces, we find that very frequently the insiduous advance has been such that the patient was unaware of the abnormal condition, or, being conscious of it, does not seek the services of a dentist until a fracture occurs or pain ensues.

It is then necessary to remove the cause of odontalgia—to get rid of an impingement upon the domain of vitality—

to take out the decayed portion, and, when the parts have been restored to a physiological condition, then cap that exquisitely sensitive and highly organized, complicated arrangement of nerve, artery, vein and connective tissue—the pulp—and finally fill the cavity produced by caries.

Where there is considerable irritation of pulp tissue, and where inflammation or congestion follows, it dies, or if the case then presents for treatment, its devitalization should be completed, and when in proper condition, the space occupied by the pulp before its extirpation and the cavity of decay should be filled. When one possesses the ability to treat *successfully*, and perform the operation of placing a material in a cavity so as to best supply the place of the lost tooth substance *artistically*, in cases where such complication exists, then one can readily succeed in those more simple. When cavities occur where the surfaces of the teeth approximate, presenting quite an extent of ruin, compound fillings are made necessary, and for the durability of teeth so affected, desirable.

In the April number of the *Dental Cosmos*, 1871, Dr. Louis Jack, of Philadelphia, described the manner of using the matrices designed by himself to simplify the filling of such cavities in bicuspids and molars. These matrices have been adopted by many, but as they are to be used after teeth are separated by pressure, and are so shaped that the contour of the tooth would be restored when the filling was completed, and now that a number of practitioners are accepting wholly, or in part, the views of Dr. Robert Arthur, of Baltimore, and are permanently separating the teeth, these appliances do not come into requisition so frequently.

Those who have been practicing the restoration of such contour and who have not, or only partially, adopted the views set forth in the recent work on the "*Treatment and Prevention of Decay of Teeth*," will continue the use of such excellent ideas. Cases present, not perhaps in the practice of every individual, but very often, where separation by pressure cannot conveniently be affected, or where it is best not to do so, sufficiently to admit of the application of a matrix of this character, then some modification and different manipulating may be required. Sometimes, too, no contour filling is indicated, because of the formation of a small cavity with firm lateral walls, though requiring a compound filling. In such cases a very simple, yet a very convenient and excellent aid is a portion of a fine separating file, such as Froid *oo*.

These can be placed in position when teeth are quite closely in contact; indeed, often as much as the thickness of such an appliance should be trimmed from the frail edges of the carious portion of a tooth, and sufficient space is thereby gained. To retain this matrix, the fine file-cut surface may be placed next the cavity, and will assist to secure such matrix when introducing the wedge between its smooth surface and the adjoining tooth, and while impacting the material for filling whether in the form of cylinders, blocks, pellets, etc., or gold or tin foil. When the filling is inserted the surface will not be so smooth as if the matrix invented by Dr. Jack were used; however, some finishing is always necessary, no matter what may be the matrix employed, or plan adopted. Where the proximal surfaces of either bicuspids or molars have been so attacked by caries as to cause the almost complete breaking up of such surfaces, and the walls are attenuated, it is best to cut away the overhanging edges of enamel, and gain free access from the masticating surface.

After applying the rubber dam, trimming away that portion of the margin which is likely to fracture, removing the decayed part, cutting out the fissures and making the cavity somewhat larger within than the outer edges, so as to retain the filling, apply a matrix, and the cavity is thus made a simple one.

When two teeth are thus attacked by caries where they come into apposition, the cavity in the one should be well prepared, filled, and the filling finished, and then the other.

Restore the proper contour of both, so that when the teeth approximate, the most prominent portion of the fillings will come in contact, and that the entire margin, against which the gold is packed, may be free, thus making the parts self-cleansing from the action of the muscles of the cheek and tongue. No dentine is then exposed, and if the operation be thoroughly completed, caries will not often recur, even if due cleanliness be not observed, which observance is absolutely necessary, for a considerable time at least, when permanent separations, requiring removal of enamel and often exposure of dentine, are practiced successfully. Maintain the same care in keeping the proximal surfaces of teeth cleansed and polished from their eruption without permanent separation, as must be done after the same is accomplished, and the enamel, as naturally presented, will most assuredly not yield to destructive agents, but become more dense as years pass by, and caries will thus be prevented. When cavities present in the proximal surfaces of bicuspids or molars where a considerable portion has been destroyed, it is quite frequently necessary to cut away some of the attenuated buccal as well as the palatal wall of enamel. In such case it is best to restore the contour, and allow the tooth, when filled, to come into apposition with the next in the arch at the curvature of the base of the buccal cusp thus built out. A separation is thus made toward the necks of the teeth as in nature, and as caries frequently extend along the dentine underneath the plate of enamel, so as to involve the whole of the proximal surface to the cementum, and the latter possessing the vitality to resist decay, it is quite certain that those parts will not again suffer from an attack of destructive agents, if the operation of preparing and filling the cavity and restoring the contour be thoroughly completed. The food, which may be pressed toward the gum during mastication, on entering the v-shaped space thus made, is, or can be, readily dislodged, but when forced between the parts of the teeth in apposition when the double v-separation is made, it is often retained at the necks of the teeth, and if not removed, caries may ensue, such removal, at times, being difficult for the patient to accomplish. When these v or double v-separations are made, it is said to be done that the surfaces may be self-cleansing. So far as particles of food, large enough to be detected by the tongue, are concerned, this is true; so also, will the same prove true where no separation is made—the presence of such particles will be manifested through the pressure exerted, when wedged between the teeth, even if ever so slight, and no comfort will be afforded until such cause be removed. It is the atoms—the sediment—or food, collecting upon surfaces where the almost constant friction of the tongue and cheek is not had, which are acted upon, so as to assist in the causation of caries.

The tongue can remove none such from those separated surfaces; so that, if cleanliness be maintained, this being essential to prevent a recurrence of decay, it must be by artificial means, which will be more effective if permanent separations be not resorted to. When these separations are made by, or the case is in the care of an unskillful, or careless operator, or where the patient is one who will not preserve the cleanliness necessary, one can readily foresee fail ure. These failures will thus occur from time to time, and frequently until they will outnumber those from first-class operations in ordinary cavities without permanent separation, or from good, solid, artistic contour fillings.

When caries attack the proximal surfaces of incisor or cuspid teeth, and cause a fracture of the labial and palatal walls, the filling of such cavities is made difficult. The difficulty can frequently be overcome by taking a fine file and placing it between the teeth, with the file-cut surface resting on the adjoining tooth, the other side pressed against the palatal wall of the one to be filled, and held there firmly with the fingers of the left hand placed on the extension of the file. The filling is then introduced around the gold screw, or screws, or into the retaining points or grooves, and against the matrix, until the whole, or a part is completed. Where a portion of both the labial and palatal plates of enamel are gone the same matrix may be used by the bridging over, as it were, the space between the file and palatal margin of the cavity. When the remainder is filled and the matrix removed, the unconsolidated portion of gold on the palatal surface can be picked off, or the whole made compact, and the part fully built out.

When decay is so advanced as to necessitate the trimming away and filing from another than the proximal surface, the correct practice is to sacrifice the palatal edge of enamel. The same style of matrix serves a good purpose here also, so that when the enamel is broken down to near the cutting edge of the tooth, and is removed to secure firm edges and to enable one to apply the force in packing the gold in line with the tooth, thus lessening the danger of fracturing the labial wall, such matrix can sometimes be applied much on the same principle as for the bicuspids and molars. There are cases where the palatal wall of an incisor or cuspid tooth has not been affected by caries sufficiently to cause any injury to the enamel, while that of the labial surface is very brittle and often fractured, the opaque decay being clearly visible.

Very many practitioners object to exposure of gold, and rightly so too, where the exposure can possibly be avoided, but what shall be done when such a case as this presents? Resort to extraction? No, never, so long as a root remains with its cementum in a normal condition. Place crowns on such roots by first-class operations known as "pivoting," or otherwise. If quite a number of roots remain, or if some are missing from the anterior alveolar process, then extraction and the adaptation of teeth on a plate may be advisable. The best one can do when a case presents where the labial wall of enamel is fractured, or a portion of an incisor or cuspid tooth is lost, is to make the edges regular, restore the proper contour with a solid gold filling, and finish perfectly, when, with all indentations obliterated, such a filling will present an appearance as if burnished, though under some circumstances, this burnishing should be dispensed with. Such an operation *well performed* is decidedly preferable to that where quite a space remains in consequence of the cutting away of tooth substance, often because of inability to perform a really good, artistic operation, but professedly to avoid the exposure of gold, while at the same time a lateral view reveals quite as much as otherwise, and the unnecessary space remains to mar the appearance not only of the other teeth, but of the face.

---

